# The Functional Study of a Chinese Herbal Compounded Antidepressant Medicine – Jie Yu Chu Fan Capsule on Chronic Unpredictable Mild Stress Mouse Model

**DOI:** 10.1371/journal.pone.0133405

**Published:** 2015-07-17

**Authors:** Lingling Ding, Xiaoyu Zhang, Hongliang Guo, Junliang Yuan, Shujuan Li, Wenli Hu, Teresa Golden, Ning Wu

**Affiliations:** 1 Department of Neurology, Beijing Chaoyang Hospital, Capital Medical University, Beijing, China; 2 Department of Biological Sciences, Southeastern Oklahoma State University, Durant, Oklahoma, United States of America; Xi'an Jiaotong University School of Medicine, CHINA

## Abstract

Jie Yu Chu Fan capsule (JYCF) is a new compounded Chinese herbal medicine for the treatment of depression. The present study was designed to explore the antidepressant effects and the possible mechanisms of JYCF by using chronic unpredictable mild stress (CUMS) mouse model and comparing results to that of fluoxetine. Behavioral tests including an open field test, sucrose preference test and forced swim test were performed to evaluate the antidepressant effects of JYCF. The concentrations of monoamine neurotransmitters and metabolic products including norepinephrine (NE), 5-hydroxytryptamine (5-HT), dopamine (DA), 5-hydroxyindoleacetic acid (5-HIAA), homovanillic acid (HVA) and 3,4-dihydroxyphenylacetic acid (DOPAC) in the cerebral cortex and hippocampus of mice were determined by means of high performance liquid chromatography with electrochemical detection (HPLC-EC). The results show that a successful mouse CUMS model was established through 5 weeks of continuous unpredictable stimulation, as indicated by the significant decrease in sucrose preference and locomotor activity and increase in immobility time in the forced swim test. Chronic treatment of JYCF (1.25, 2.5 and 5 g/kg) and fluoxetine (20mg/kg) significantly reversed the CUMS-induced behavioral abnormalities. JYCF (1.25, 2.5 and 5 g/kg) significantly increased NE in CUMS mouse prefrontal cortex (P < 0.01, P < 0.01, P < 0.05 respectively) and 5-HT in hippocampus (P < 0.05). In summary, our findings suggest that JYCF exerts comparable antidepressant-like effects to that of fluoxetine in CUMS mice. Besides, the antidepressant-like effect of JYCF is mediated by the increase of monoaminergic transmitters including 5-HT and NE.

## Introduction

The depressive disorders accounted for 40.5% of mental and substance use disorders, which means the global burden of mental disorders continues to be higher than that resulting from any other disease category [1, 2]. In past decades, studies demonstrated that depressive disorder is caused by the decrease in the amount of neurotransmitters such as 5-hydroxytryptamine (5-HT), norepinephrine (NE), and dopamine (DA) [3]. Medication is the major treatment for depressive disorder. Antidepressants increase the concentration of 5-HT, NE, and DA neurotransmitters in the synaptic space, thus increasing neuronal activities [3,4]. Currently, selective serotonin reuptake inhibitor (SSRIs) including fluoxetine is one of the most popular antidepressant applied in the clinic. Recent studies on some Chinese herbal medicines also demonstrated similar effects of fluoxetine in depressive disorder treatment [5–8]. However, some of these drugs have undesirable side effects. In addition, there are still a large number of people with depression in the least developed countries unable to receive effective treatment for many reasons [1]. Therefore, there is an urgent need for new effective and better tolerable antidepressant.

Jie Yu Chu Fan capsule (JYCF), composed of Gardenia jasminoides Ellis (ZZ), magnoliae officinalis (HP), Pinellia ternata Breit and Forsythia suspense, is a new compounded Chinese herbal medicine for the treatment of depression. ZZ, HP and Pinellia ternata Breit are commonly used in many traditional Chinese medicines such as Banxia-houpu decoction (BHD) and Zhizi-houpu decoction (ZZHPD), which has been used for centuries to treat mental disease including depression and other disorders [9–11]. The aim of the present study was to explore the antidepressant effects of JYCF by using CUMS mouse model and comparing it to that of fluoxetine. To further investigate the mechanism, the concentrations of monoamine neurotransmitters and metabolic products including NE, 5-HT, DA, 5-hydroxyindoleacetic acid (5-HIAA), homovanillic acid (HVA) and 3,4-dihydroxyphenylacetic acid (DOPAC) in cerebral cortex and hippocampus of mice were determined by means of high performance liquid chromatography (HPLC) with electrochemical detection.

## Materials and Methods

### 1. Ethics statement

All animal studies were conducted with the National Institute of Health Guidelines for the Care and Use of Laboratory Animals. All experiments were approved by the Comments of Animal Experiments and Experimental Animal Welfare Committee of Capital Medical University. We abide by ethical principles of animal welfare. To minimize animal suffering, the animals were sacrificed by cervical dislocation after the experiments.

### 2. Animals

Forty eight adult male Imprinting Control Region (ICR) mice weighing 20–25g were purchased from Beijing Vital River Laboratories (Beijing, China), the distributor of The Jackson Laboratory (USA). The entire animal experimental process was approved by the Comments of Animal Experiments and Experimental Animal Welfare Committee of Capital Medical University. Each individual animal was fed in single cage under the environment of room temperature 20±2°C, humidity 40–60%, and light/dark alternation in 12 hours with the light on from 7:00 am to 7:00 pm. All animals were free to access the water and food. One week of environmental adaptive feedings were applied right before the experiments.

### 3. Chronic unpredictable mild stress (CUMS) procedures

**Fig 1:**
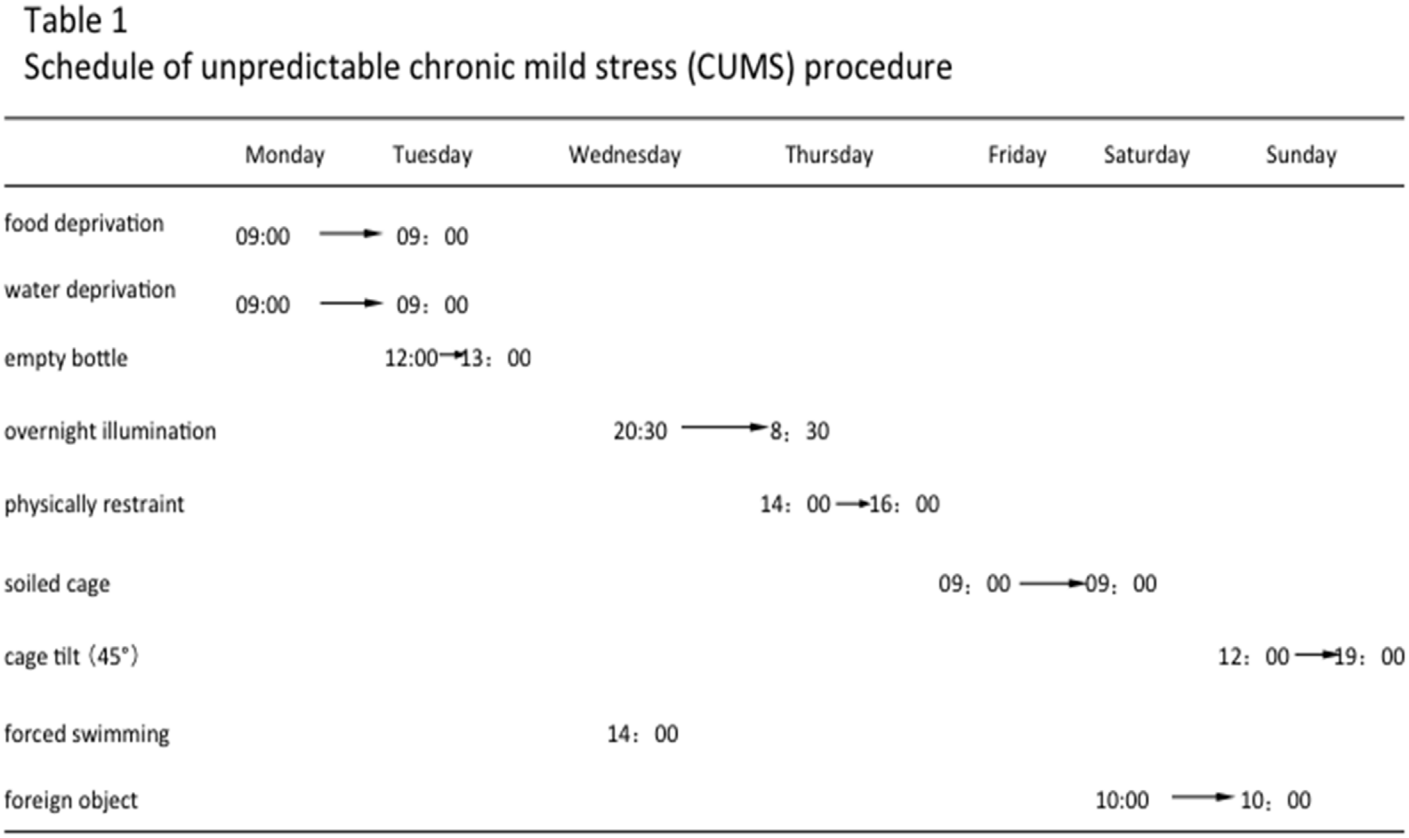
Schedule of unpredictable chronic mild stress (CUMS) procedure. The above stimulations were arranged for one random type of stimulation per day with no repeat of the same type of stimulation in continuous days, which would guarantee the animal will face unpredictable stimulation. The whole stimulation process lasted for 5 continuous weeks.

**Fig 2:**
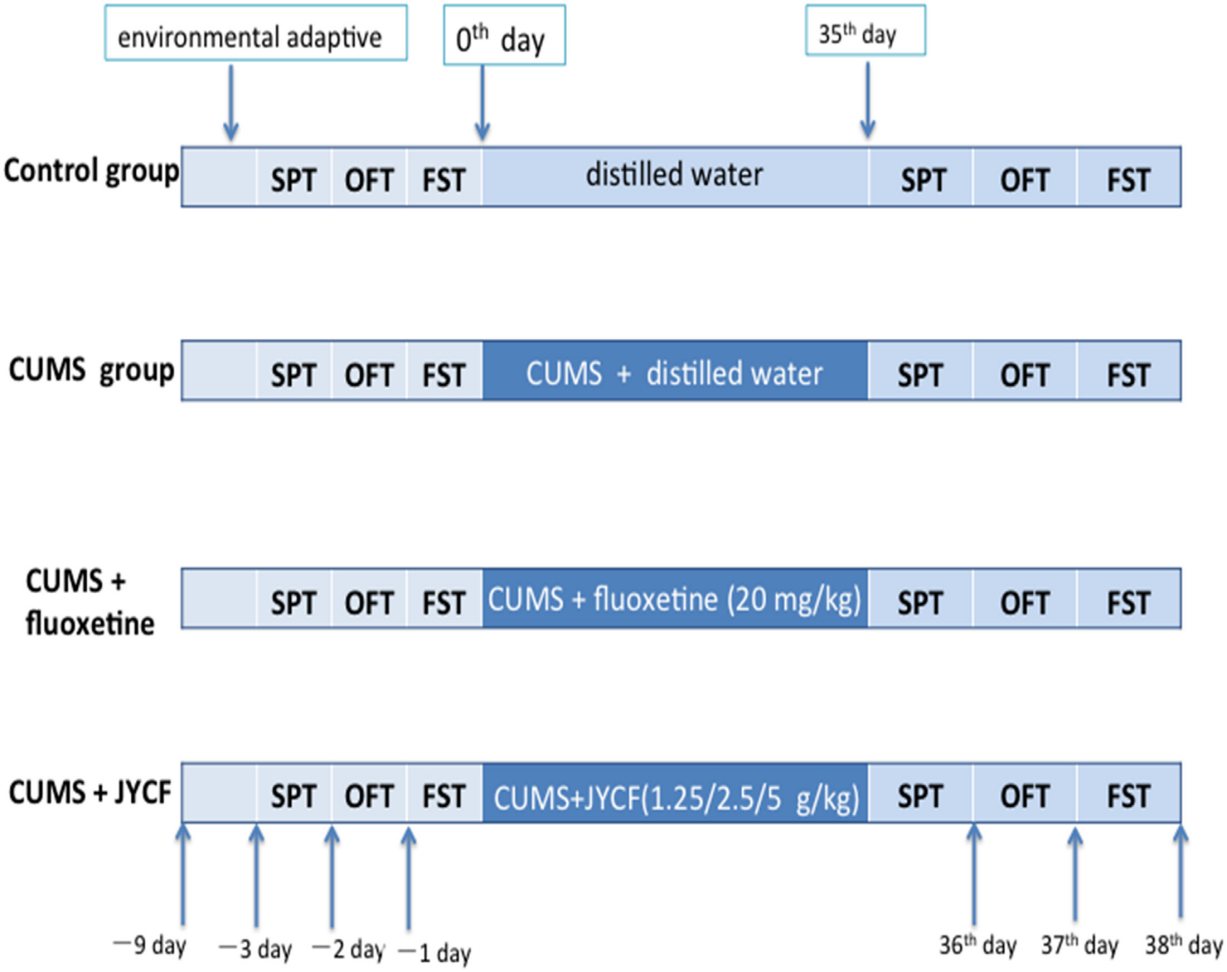
The sequence of the behavioral test. Behavioral tests including an open field test, sucrose preference test and forced swim test were performed to evaluate the antidepressant effects of JYCF.

Except the control group, all experimental animals were fed in single cages and in the same room [12]. According to previous CUMS mouse model methods, the following stimulations were applied to the experimental animals, which included (1) food deprivation for 24 hours, (2) water deprivation for 24 hours, (3) exposure to a empty bottle for 1 hour, (4) cage tilt (45 degrees) for 7 hours, (5) overnight illumination, (6) soiled cage (200 ml water in 100 gram sawdust bedding) for 24 hours, (7) forced swimming at 8°C for 6 minutes (8) physical restraint for 2 hours, and (9) exposure to a foreign object (e.g. a piece of plastic) for 24 hours [5, 13]. The above stimulations were arranged for one random type of stimulation per day with no repeat of the same type of stimulation in continuous days, which would guarantee the animal will face unpredictable stimulation (Fig 1). The whole stimulation process lasted for 5 continuous weeks. The behavioral experimental tests were performed 24 hours after the last stimulation, which included the sucrose preference test, forced swim test, and open field test(Fig 2). All animals were sacrificed 24 hours after the behavior experiments. The prefrontal cortex and hippocampus of each animal were isolated, instantly frozen in liquid nitrogen and stored at -80°C freezer.

### 4. Drug administration and experimental groups

All experimental animals were randomly divided into 6 groups with 8 animals per group including (1) Control group: non-stressed + distilled water; (2) CUMS group: CUMS + distilled water; (3) CUMS + fluoxetine (20 mg/kg); (4) CUMS + JYCF (1.25 g/kg); (5) CUMS + JYCF (2.5 g/kg); (6) CUMS + JYCF (5.0 g/kg). All medicines were dissolved in distilled water in designated concentrations. All drugs were administered orally (via intragastric gavage) once a day in the morning between 9:00 am to 10:00 am for 5 weeks without interval. The stimulations were conducted two hours after the drug treatment.

### 5. Open field test

The open field test was performed as described previously [14]. Experimental animals were placed in the center of an open box with the sizes of 45 cm each for length, height, and width. The inner surfaces of the box were painted black and 25 equal area squares were divided at the bottom of the box. The following behaviors were obtained: the number of crossings (the number of squares crossed); rearing (the frequency of standing on hind limbs). The times for both scoring measurements were 3 minutes.

### 6. Sucrose preference test

The sucrose preference test was performed as described previously [15]. Briefly, all animals were trained adaptively for 1% sucrose solution before experiments. 1% sucrose solution was applied as a substitute for the regular drinking water for experimental animals 24 hours before the experiment. After the adaptation, mice were deprived of water and food for 12 hours. Sucrose preference test was conducted at 9:00 am when mice were housed in individual cages and were free to access to two bottles containing 100 ml of 1% sucrose solution and 100 ml of sterilized distilled water, respectively. After 1 hour, the consumption volume (ml) of sucrose solution (V1) and sterilized distilled water (V2) were recorded and the preference of sucrose (%) was calculated as V1 x 100/ (V1 + V2).

### 7. Forced swim test

Forced swim test was performed as described previously [16]. The experimental animal was placed in a round glass container with the diameter and height of 20 cm x 15 cm. The water was filled to 10 cm height level with the temperature of 25 ± 5°C. The swimming experiment lasted for 6 minutes. The animal immobility time in the last 4 minutes were recorded. The criteria for animal immobility recognition were: (1) stopped struggling; (2) upright floating posture; (3) occasional limb movement to maintain the head above the water level with no observed body movement.

### 8. HPLC-EC measurements of brain monoamine neurotransmitters and metabolic products

HPLC-EC technology was applied to measure the concentrations of brain monoamine neurotransmitters 5-HT, DA, NE, and their metabolic products 5-HIAA, HVA, DOPAC respectively. In brief, animal brain tissues were mixed with 0.4 ml of pre-chilled internal labeling working solution and then were homogenized in ice-cold water bath. After standing for 10 minutes, the mixtures were centrifuged at 14,000 g for 15 minutes at 4°C. The supernatants were collected by passing the solution through a 0.2μm filter membrane. Total 100 μl of filtered sample solution was applied to HPLC-EC column of ESA MD-150 (3.2 x 150 mm) with the mobile phase of 90 mM sodium dihydrogen phosphate, 50 mM citric acid, 1.7 mM 1- octane sulfonate, 50 μM EDTA, and 10% acetonitrile. The experimental conditions were set as follows: pH: 3.0, flow rate: 0.6 ml/min, sample injection volume: 20 μl, column temperature: 30°C. Four electrical potentials were set for the experiments as -50, 150, 350, and 500 mV. ESA software was used for data analysis. The calculated neurotransmitter concentration was expressed as ng of neurotransmitter per gram of tissue.

### 9. Data analysis

Data are expressed as means ± SD. Multiple group comparisons were performed using one-way analysis of variance (ANOVA) followed by Dunnett’s test. P<0.05 was considered significant. SPSS17.0 statistical software was applied for statistical analysis.

## Results

Pre-experimental data for individual animals and each group were collected and analyzed as the control baseline information. All experimental data were collected after 5-weeks CUMS exposure.

### 1. The effects of JYCF on animal body weights

**Fig 3:**
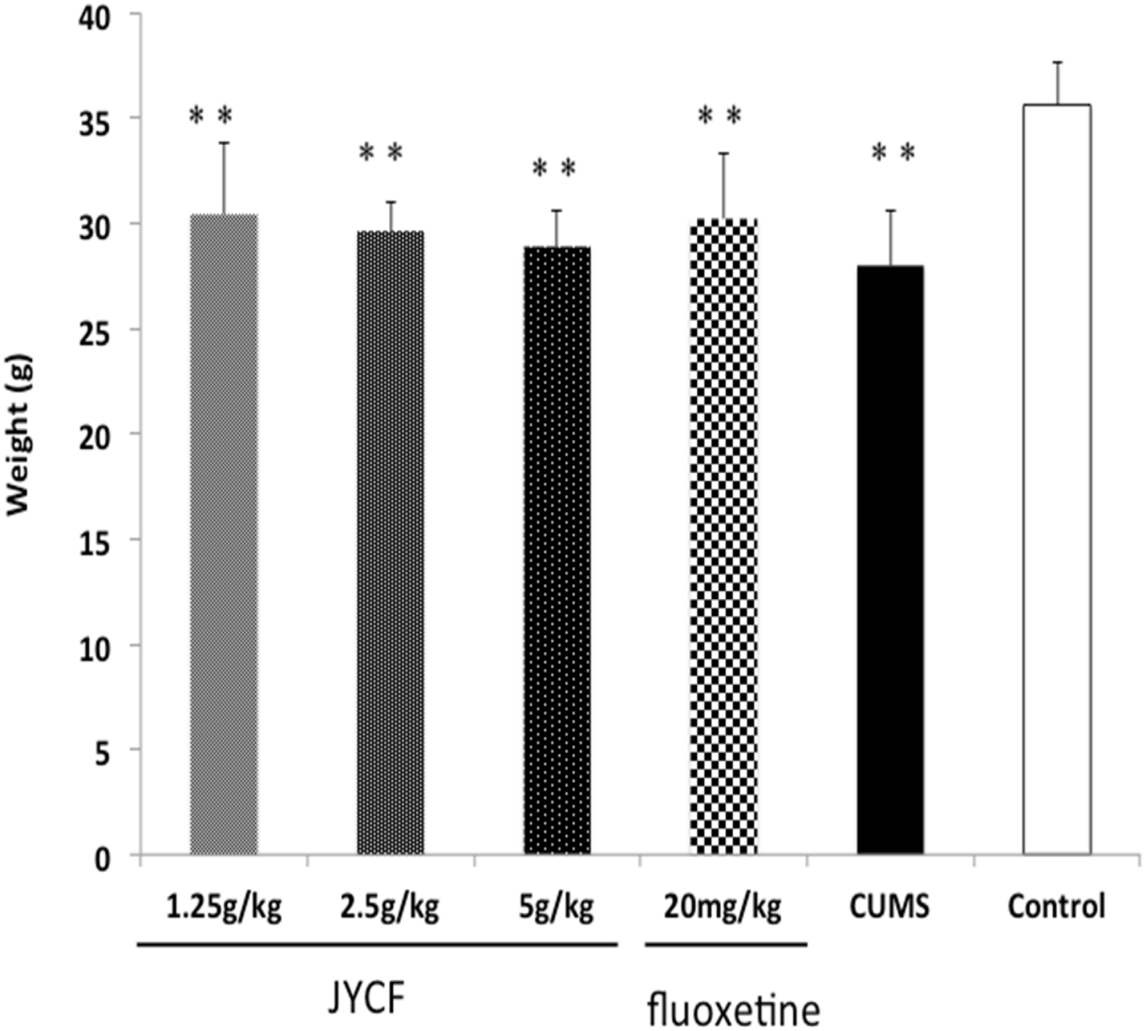
Effect of JYCF and fluoxetine on weight. A one-way ANOVA indicated there was a significant body weight loss in CUMS model groups compared to that of normal control group [F (5,42) = 9.739, P < 0.01] after 5- week CUMS exposure. The chronic treatment of CUMS mice with JYCF (1.25, 2.5, 5 g/kg) or fluoxetine (20 mg/kg) did not reverse the body weight loss caused by CUMS(P > 0.05). Results are expressed as mean ± S.E.M. (n = 8 each). **P < 0.01, as compared with the control group.

A one-way ANOVA indicated pre-experimental animal body weights were not significantly different among the groups [F (5, 42) = 0.969, P > 0.05]. After 5-week CUMS exposure, there was a significant body weight loss in CUMS model groups compared to that of normal control [F (5, 42) = 9.739, P < 0.01]. However, post hoc testing showed that chronic administration of JYCF at doses of 1.25 g/kg, 2.5 g/kg, 5 g/kg and fluoxetine (20 mg/kg) did not reverse the body weight loss caused by CUMS (P > 0.05) (Fig 3).

### 2. Effects of JYCF on open field test

**Fig 4:**
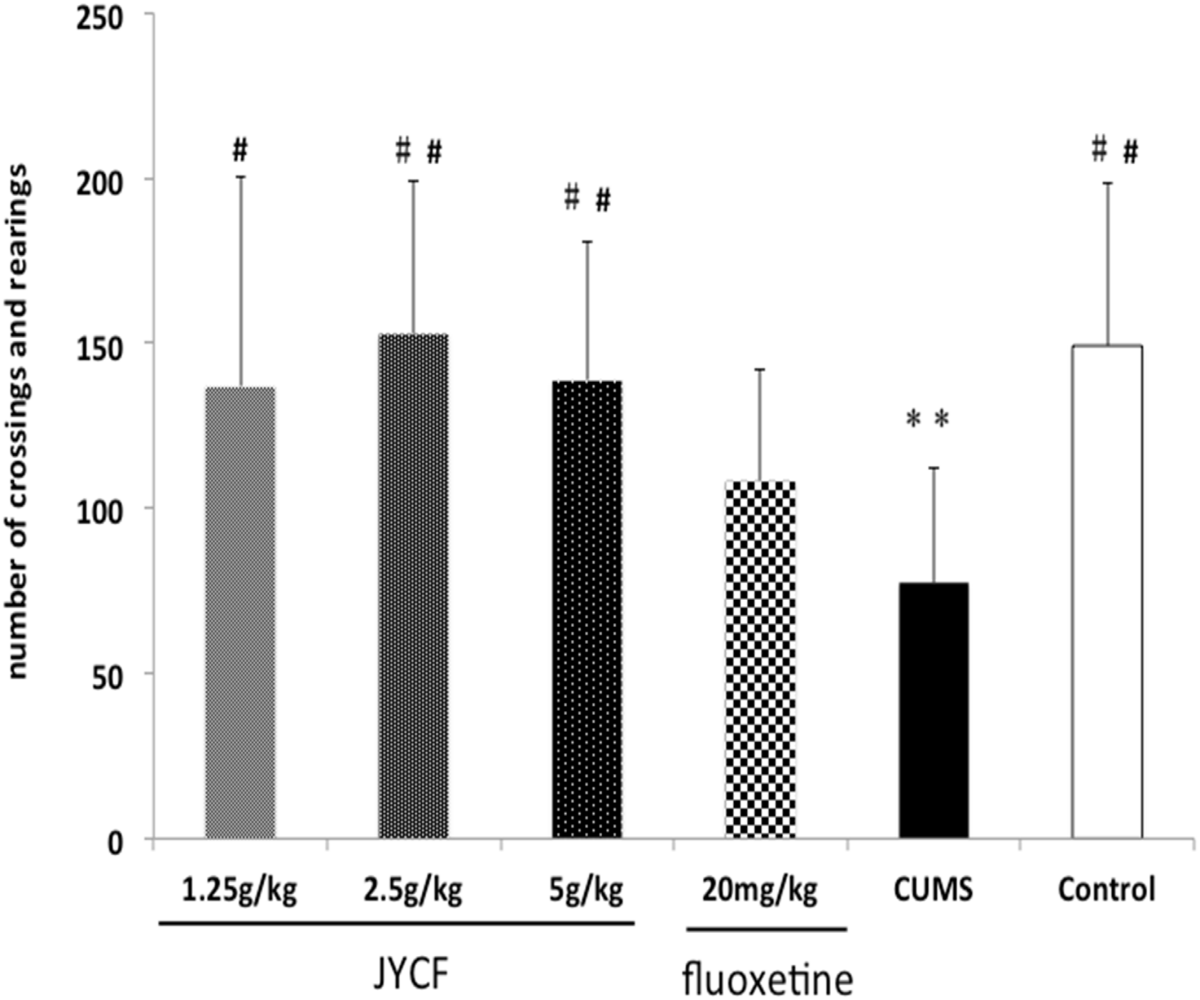
Effect of JYCF and fluoxetine on the number of crossings and rearings in the open-field test. A one-way ANOVA demonstrated a significant reduction in number of crossings and rearing in comparison to the normal control group [F (5, 42) = 3.323, P < 0.05] after 5-week CUMS exposure. Post hoc testing showed that JYCF (1.25 g/kg, 2.5 g/kg, and 5 g/kg) improved the number of crossings and rearings significanctly (P < 0.05, P < 0.01, and P < 0.01 respectively). Results are expressed as mean ± S.E.M. (n = 8 each). **P < 0.01, as compared with the control group; #P < 0.05, ##P < 0.01, as compared with the CUMS group.

The one-way ANOVA showed there were no significant differences in the number of crossings and rearings among the six groups [F (5, 42) = 0.633, P > 0.05] at the beginning of the experiment. After 5-week CUMS exposure, the open field test demonstrated that there was a significant reduction in the number of crossings and rearings comparing to the normal control group [F (5, 42) = 3.323, P < 0.05]. Post hoc testing showed that JYCF treatment groups at the dose of 1.25 g/kg, 2.5 g/kg, and 5 g/kg improved the number of crossings and rearings significantly (P < 0.05, P < 0.01, and P < 0.01 respectively) (Fig 4).

### 3. Effect of JYCF on immobility time in forced swim test

**Fig 5:**
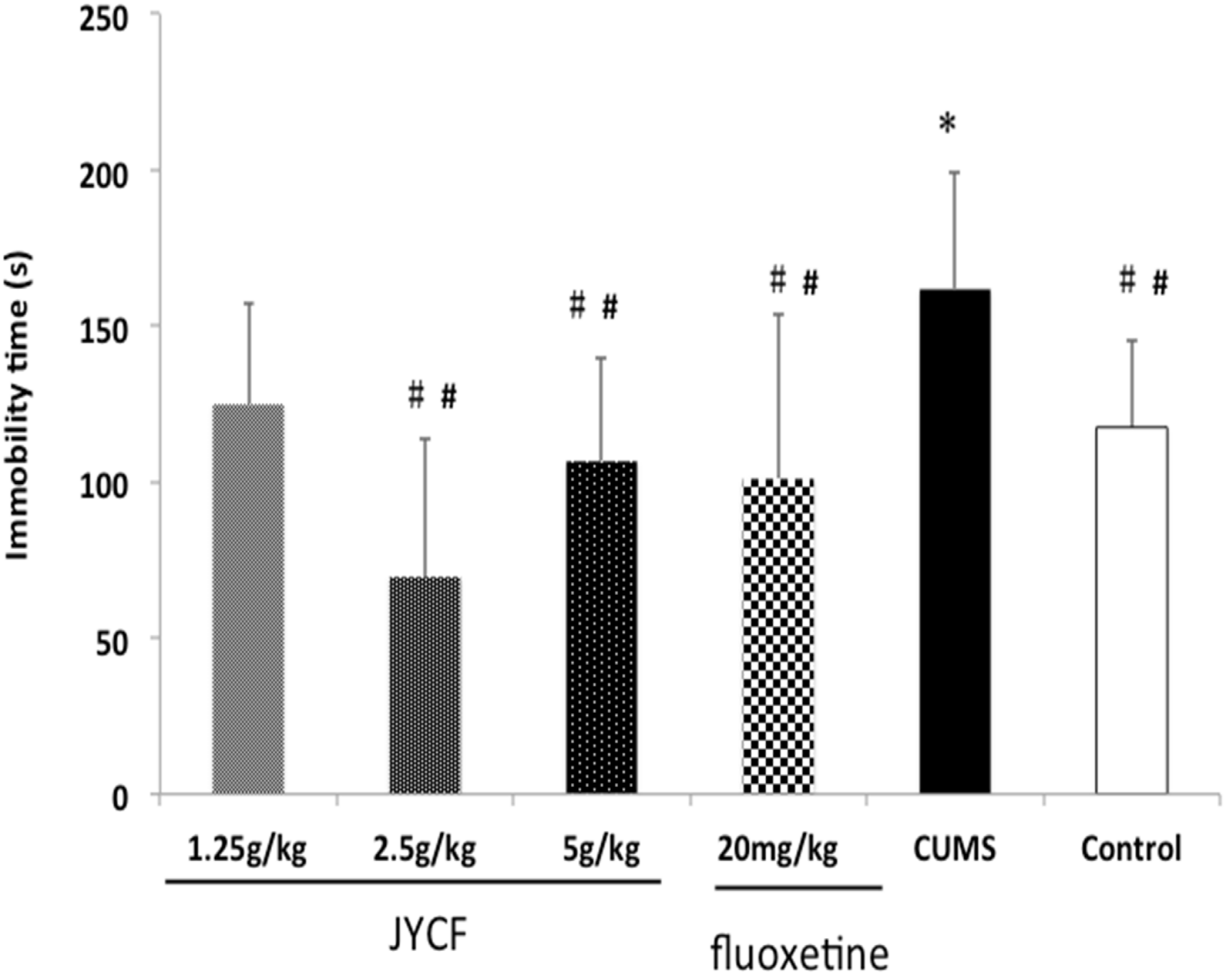
Effect of JYCF and fluoxetine on the immobility time in the forced swimming test. The one-way ANOVA showed significantly increased immobility time in the forced swimming test in comparison with the control group [F (5, 42) = 4.825, P = 0.001] after 5-week CUMS exposure. Post hoc testing showed that 2.5 g/kg and 5 g/kg JYCF could significantly reduce the immobility time comparing to CUMS group (P < 0.01). Results are expressed as mean ± S.E.M. (n = 8 each). *P < 0.05, as compared with the control group; ##P < 0.01, as compared with the CUMS group.

The one-way ANOVA showed there were no significant differences in immobility time among the six groups [F (5, 42) = 0.539, P > 0.05] at the beginning of the experiment. After 5-week CUMS exposure, the immobility time was remarkably increased compared to the normal control group [F (5, 42) = 4.825, P = 0.001]. Post hoc testing showed that 2.5 g/kg and 5 g/kg JYCF could significantly reduce the immobility time compared to CUMS group (P < 0.01). The positive control fluoxetine (20 mg/kg) also significantly shortened the immobility time compared to the CUMS group (P < 0.01) (Fig 5).

### 4. Effect of JYCF on the percentage of sucrose consumption

**Fig 6:**
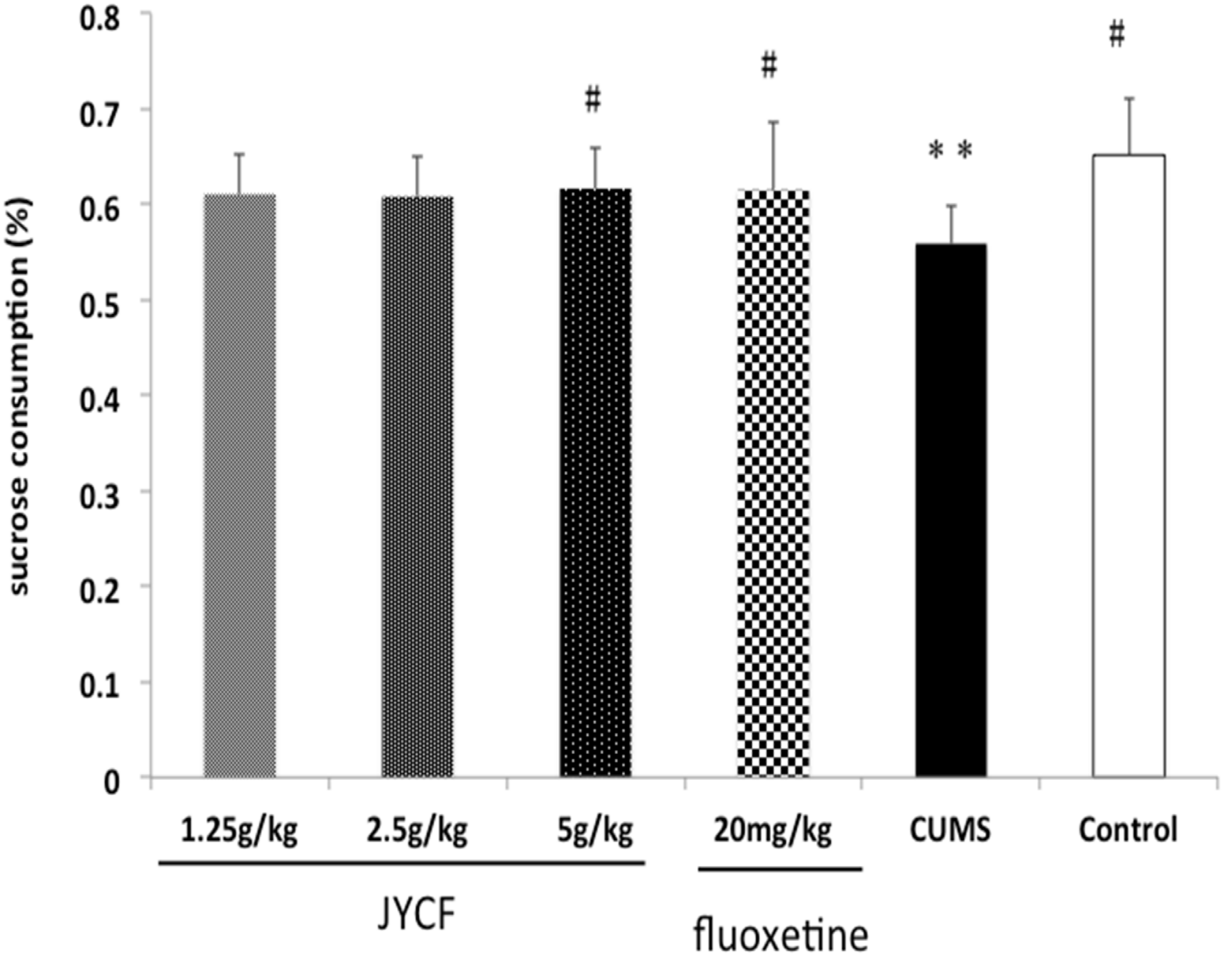
Effect of JYCF and fluoxetine on the sucrose consumption test. A one-way ANOVA indicated significantly decreased the percentage of sucrose consumption comparing to that of control group [F (5, 42) = 2.703, P < 0.05] after 5-week CUMS exposure. Post hoc test showed that JYCF (5 g/kg) and fluoxetine (20 mg/kg) significantly reversed the reduction in the percentage of sucrose consumption as compared to the CUMS group (P < 0.05). Results are expressed as mean ± S.E.M. (n = 8 each). **P < 0.01,as compared with the control group; #P < 0.05, as compared with the CUMS group.

A one-way ANOVA indicated there was no significant difference among the pre-experimental animal groups [F (5, 42) = 0.367, P > 0.05]. After 5-week CUMS exposure, the sucrose consumption of CUMS mice was significantly reduced to that of the normal control group [F (5,42) = 2.703, P < 0.05]. Post hoc test showed that JYCF at dose of 5 g/kg showed a significant preference of sucrose comparing to that of CUMS group (P < 0.05). The positive control fluoxetine (20 mg/kg) also significantly reversed the reduction in the percentage of sucrose consumption as compared to the CUMS group (P < 0.05) (Fig 6).

### 5. Brain concentrations of monoamine neurotransmitters and metabolic products

**Fig 7:**
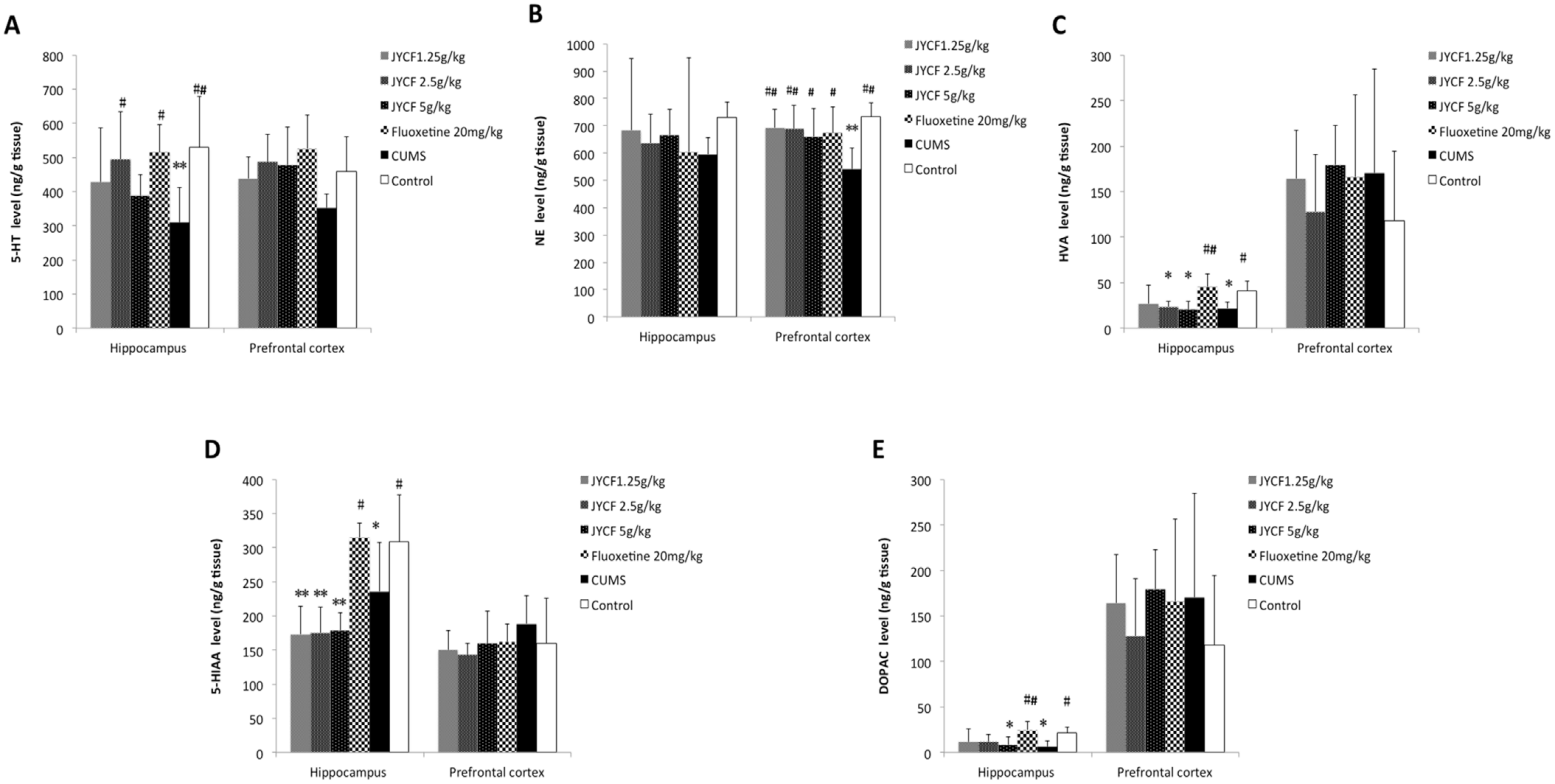
Effect of JYCF and fluoxetine on the levels of brain monoamine neurotransmitters and metabolic products in mice. **(A)** The levels of 5-HT in the hippocampus and prefrontal cortex. In the hippocampus, one-way ANOVA indicated a significant effect [F (5, 42) = 3.037, P < 0.05] of treatment with JYCF and fluoxetine on 5-HT concentration. JYCF (2.5 g/kg) and fluoxetine (20 mg/kg) significantly increased 5-HT levels compared to the CUMS group (P < 0.05). In the prefrontal cortex, one-way ANOVA indicated no significant effect [F (5, 42) = 2.347, P > 0.05]. Results are expressed as mean ± S.E.M. *P<0.05, **P < 0.01, as compared with the control group; #P < 0.05, ##P < 0.01, as compared with the CUMS group. (B) The levels of NE in the hippocampus and prefrontal cortex. In the prefrontal cortex, a one-way ANOVA revealed a significant effect [F (5, 42) = 3.125, P < 0.05] of treatment with JYCF and fluoxetine on NE concentration. JYCF (1.25, 2.5, 5g/kg) and fluoxetine (20 mg/kg) significantly increased NE level compared to the CUMS group (P < 0.01, P < 0.01, P < 0.05, P< 0.05 respectively). In the hippocampus, one-way ANOVA indicated no significant effect [F (5, 42) = 0.359, P > 0.05]. Results are expressed as mean ± S.E.M. *P<0.05, **P < 0.01, as compared with the control group; #P < 0.05, ##P < 0.01, as compared with the CUMS group. (C) The levels of HVA in the hippocampus and prefrontal cortex. In the hippocampus, a one-way ANOVA indicated significantly difference after administration of JYCF and fluoxetine in CUMS mice on the concentration of HVA [F (5, 42) = 4.070, P < 0.01], JYCF (1.25, 2.5, 5 g/kg) did not reverse the reduction of HVA (P > 0.05). In the prefrontal cortex, one-way ANOVA indicated no significant effect [F (5, 42) = 0.411, P > 0.05]. Results are expressed as mean ± S.E.M. *P < 0.05, **P< 0.01, as compared with the control group; #P < 0.05, ##P < 0.01, as compared with the CUMS group. (D) The levels of 5-HIAA in the hippocampus and prefrontal cortex. In the hippocampus, a one-way ANOVA indicated significantly difference after administration of JYCF and fluoxetine in CUMS mice on the concentration of 5-HIAA [F (5, 42) = 9.783, P < 0.01], JYCF (1.25, 2.5, 5 g/kg) did not reverse the reduction of 5-HIAA (P > 0.05). In the prefrontal cortex,one-way ANOVA indicated no significant effect [F (5, 42) = 0.740, P > 0.05]. Results are expressed as mean ± S.E.M. *P < 0.05, **P < 0.01, as compared with the control group; #P < 0.05, ##P < 0.01, as compared with the CUMS group. (E) The levels of DOPAC in the hippocampus and prefrontal cortex. In the hippocampus, a one-way ANOVA indicated significantly difference after administration of JYCF and fluoxetine in CUMS mice on DOPAC [F (5, 42) = 3.291, P < 0.05], JYCF (1.25, 2.5, 5 g/kg) did not reverse the reduction of DOPAC (P > 0.05). In the prefrontal cortex, one-way ANOVA indicated no significant effect [F (5, 42) = 0.530, P > 0.05]. Results are expressed as mean ± S.E.M. *P < 0.05, **P < 0.01, as compared with the control group; #P < 0.05, ##P < 0.01, as compared with the CUMS group.

In the hippocampus, a one-way ANOVA indicated a significant effect [F (5, 42) = 3.037, P < 0.05] of treatment with JYCF and fluoxetine on 5-HT concentration. Post hoc testing showed that 2.5 g/kg JYCF and fluoxetine at 20 mg/kg significantly increased 5-HT levels compared to the CUMS group in the hippocampus (P < 0.05). In the prefrontal cortex, one-way ANOVA indicated no significant effect of treatment with JYCF and fluoxetine on 5-HT concentration [F (5, 42) = 2.347, P > 0.05] (Fig 7A). In the prefrontal cortex, a one-way ANOVA revealed a significant effect of treatment with JYCF and fluoxetine on NE concentration [F (5, 42) = 3.125, P < 0.05]. The Dunnett’s post hoc analysis indicated a significant increase in NE level in the prefrontal cortex of mice with the administration of JYCF at doses of 1.25 g/kg, 2.5 g/kg, 5 g/kg and fluoxetine at doses of 20 mg/kg, when compared to the CUMS group (P < 0.01, P < 0.01, P < 0.05, P < 0.05 respectively). In the hippocampus, one-way ANOVA indicated no significant effect of treatment with JYCF and fluoxetine on NE concentration [F (5, 42) = 0.359, P > 0.05] (Fig 7B). JYCF and fluoxetine did not exhibit significant changes in the concentration of DA both in prefrontal cortex and hippocampus.

In the hippocampus, a one-way ANOVA indicated a significant difference after administration of JYCF and fluoxetine in CUMS mice on the concentration of HVA [F (5, 42) = 4.070, P < 0.01], 5-HIAA [F (5, 42) = 9.783, P < 0.01] and DOPAC [F (5, 42) = 3.291, P < 0.05]. The results showed that the chronic administration of JYCF at doses of 1.25 g/kg, 2.5 g/kg, 5 g/kg did not reverse the reduction of HVA, 5-HIAA and DOPAC (P > 0.05). However, fluoxetine (20 mg/kg) significantly increased the level of HVA, 5-HIAA and DOPAC in hippocampus (P < 0.01, P < 0.05, P < 0.01, respectively). There was no significant difference in the concentration of HVA, 5-HIAA and DOPAC among groups in prefrontal cortex (P > 0.05) (Fig 7C–7E).

## Discussion

Chronic unpredictable mild stress (CUMS) model firstly established by Katz and Willner has been widely used as an animal model of depression for the study of antidepressants [17–20]. Our results showed that a successful CUMS model in mice was established through 5 weeks continuous unpredictable stimulation, as indicated by the significant decrease in sucrose preference and locomotor activity and increase in immobility time in the forced swim test, which was according with the results of previous studies [21, 22].

In the present study, we found that chronic administration of JYCF or fluoxetine significantly reversed behavioral changes, presenting an antidepressant-like effect in the CUMS model of depression. Anhedonia is one of the core symptom of major depressive disorder (MDD), which reflected by a reduced sucrose preference in CUMS mice[19, 23]. Chronic administration with JYCF caused a significantly increased consumption of sucrose solution and shorten the immobility time, which is a criterion for antidepressant effects evaluation in the forced swimming test [24]. In the open field test, CUMS animals treated by JYCF presented an improvement in the locomotor and exploratory behaviors [18, 25, 26]. CUMS mice are characterized by reduction of food intake and loss of body weight, which are similar to that in patients with MDD[27]. In the present study, fluoxetine and JYCF did not reverse the body weight loss caused by CUMS, which is not in line with previous reports [6, 8, 10]. Several reasons can be taken into account, including fasting treatments during the experimental process and experimental animal individual differences. Taken together, results obtained from behavioral studies indicated that JYCF treatment produced an antidepressant-like action in CUMS-induced depression model in mice.

Several studies have suggested an important role of monoaminergic transmitters in the response to stress and modulation of depression [3, 28]. The monoamine hypothesis is a biological hypothesis stating that depressive symptoms arise from a depletion in the levels of serotonin, NE, and/or DA in the central nervous system. It is widely acknowledged that the decrease of 5- HT and NE levels in different brain areas are commonly observed both in animals and patients experiencing stress and depression, while drugs that are acting by increasing the bioavailability of monoaminergic transmitters, such as selective 5-HT reuptake inhibitors and monoamine oxidase inhibitors, are widely used in clinical depression treatment [29, 30]. Thus, to support the hypothesis that the antidepressant-like effect of JYCF is mediated by the increase of monoaminergic transmitters, the concentrations of monoamine neurotransmitters and metabolic products were determined. We paid special attention to the hippocampus and frontal cortex, which are brain regions structurally and functionally affected by stress responses and critically involved in the regulation of mood and learning function [3, 31]. In the present study, CUMS mice had remarkable low levels of NE in prefrontal cortex area and 5-HT in hippocampus comparing to that of normal control animals. Both JYCF and fluoxetine could significantly increase NE in CUMS mice prefrontal cortex and 5-HT in hippocampus. However, DA level did not significantly change in CUMS-induced depression mice. Neither JYCF nor fluoxetine affected DA levels in the animal brains. The results of this study showed that JYCF exerted antidepressant effect by regulating the level of monoamine neurotransmitter in hippocampus and prefrontal cortex of CUMS mice, particularly 5-HT and NE.

Fluoxetine is a selective serotonin re-uptake inhibitor (SSRI), which is considered as the first-line treatment for depression [32]. Fluoxetine has been seen to increase the concentration of 5-HT in patients with MDD [33,34]. Previous studies suggested that chronic fluoxetine treatment reversed CUMS-induced depressive-like behavior as well as induced a persistent increase in 5-HT levels in brain regions, such as the hippocampus and frontal cortex [32]. In the present study, fluoxetine was used as a standard antidepressant. Our results showed that the antidepressant-like effect of JYCF on the behavioral and monoaminergic transmitters was comparable to that of fluoxetine.

Jie Yu Chu Fan capsule (JYCF) is composed of Gardenia jasminoides Ellis (ZZ), magnoliae officinalis (HP), Pinellia ternata Breit and Forsythia suspense. JYCF and BHD have the similar chemical components. BHD is a traditional Chinese medicinal empirical formula widely used in therapy for depression, consisting of HP, Pinellia ternata,Perilla frutescens, Poria cocos and Zingiber officinale[10]. The polysaccharides from the BHD have been found to have the ability to reduce the duration of immobility in the TST and FST while increase 5-HT and DA levels in whole mouse brain[10,35,36]. However, BHD produced no significant increase in NE concentrations[37]. G. jasminoides Ellis, one of the other component of JYCF has exerted an ability to increase the concentration of NE and 5-HT in CUMS rat hippocampus[38,39]. In addition, research has shown that chronic stress could increase the reactive oxygen species generation (ROS) in several brain areas and regulate mood [40, 41]. Forsythia suspense, one of the components of JYCF, had been reported to have the ability to prevent ROS generation, which may contribute to the etiology and progression of major depression [42, 43]. Traditional Chinese medicines are mainly mixtures of varying herb medicines. “Synergistic interactions” is an important idea in Chinese herbal medicine, which means plant extracts contain ingredients potentiating each other's effect. It is suppose that therapeutic regimens comprising numerous drugs with unique but relevant mechanisms can usually enlarge the impact of treatment of each component, result in best efficacy with less adverse effects [44]. JYCF is a new compounded Chinese herbal medicine. Our results showed that both 5-HT and NE systems were considered to play important roles in the anti-depression effect of JYCF. And the antidepressant actions of JYCF might be mediated by “Synergistic interactions”.

In the present study, JYCF did not reverse the reduction of metabolic products including 5-HIAA, HVA and DOPA both in the hippocampus and prefrontal cortex. The results agreed with the previous study that Banxia-houpu decoction produced no significant increase in metabolic products such as 5-HIAA concentrations [35,36]. On the other hand, the ethyl acetate fraction of G. jasminosides, a major component of JYCF, could inhibited the activities of both monoamine oxidase-A (MAO-A) and monoamine oxidase-B (MAO-B) [45]. Future study is needed to investigate the underlying mechanisms of JYCF on the neurotransmitter system.

## Conclusion

Jie Yu Chu Fan capsule (JYCF) is a new compounded Chinese herbal medicine for the treatment of depression. Our result showed that JYCF exerts comparable antidepressant-like effects to that of fluoxetine in CUMS mice. 5-HT and NE systems are essential mediators for the antidepressant-like effect of JYCF in CUMS mice. Further researches are needed to explore the possible mechanism of JYCF on the neurotransmitter system and other aspects.
